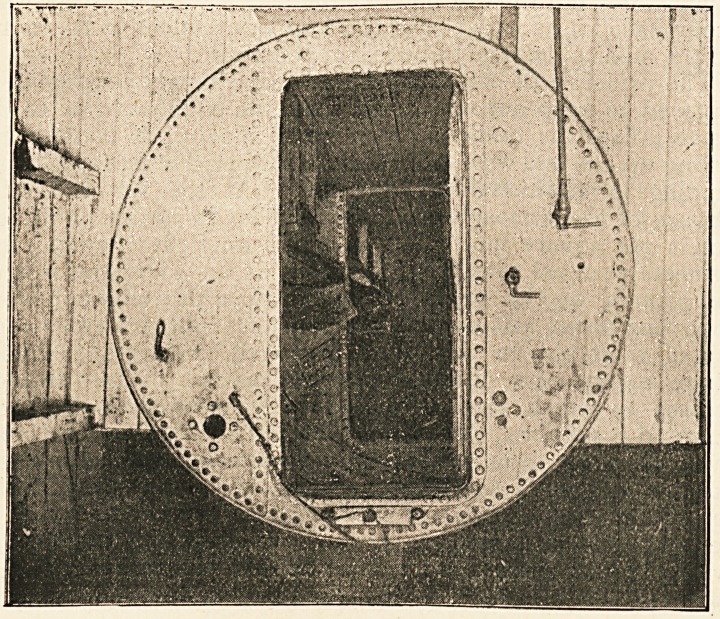# Compressed Air Illness, or So-Called Caisson Disease

**Published:** 1897-03

**Authors:** 


**Compressed Air Illness, or so-called Caisson Disease.** By E. HUGH SNELL, M.D., B.Sc. Pp. viii., 251. London: H. K. Lewis. 1896.

The London County Council made a wise selection in appointing Dr. Snell to take charge of the men employed in the compressed air work carried on at the Blackwall Tunnel. What that work was is well described and illustrated in a paper by Mr. J. M. Bulloch in the January number of the *English Illustrated Magazine*, in which diagrams show the general arrangement of "the most wonderful tunnel in the world"; it is twenty-seven feet in diameter, and has been carried through gravel ground so dangerous that the most experienced engineers deprecated the idea of its possibility. The story of the making of this tunnel is quite a scientific romance, but our duty is not so much with the engineers who accomplished it as with the medical superintendent of the work and the medical lessons to be learnt therefrom.

The principle of compressed air is exceedingly simple—it is employed to keep out water; so what the engineers had to do was to inflate the tunnel, as you would blow up a bladder, by corking up one end with a huge brick wall twelve feet thick and pumping in additional atmospheres. At the sea-level the pressure of the atmosphere is well known to be about fifteen pounds per square inch; but into this air-tight chamber the engineers pumped sometimes as much as an additional thirty-five pounds to the square inch, so that the workers were supporting about half a hundredweight of air-pressure on every inch of their bodies. Dr. Snell has well utilised the almost unique opportunity of observing the nature of the strange illnesses to which men are liable when working under such abnormal conditions, and this volume gives us by far the most advanced monograph which has yet appeared on the subject. After an outline of the history of working in compressed air, the author gives a series of fifty cases, selected from a list of over two hundred similar ones of very varying degrees of severity which have occurred under his observation at the Blackwall Tunnel, and he then gives an excellent summary of the symptoms caused by compressed air. It has been commonly assumed that some form of paraplegia is the most likely result of abnormally high air pressures. Dr. Walter Moxon's Croonian Lectures on the cerebral circulation (*Lancet*, 1881) gave a very good anatomical reason why the spinal cord should suffer from anæmia in consequence of the length and smallness of the arteries in the long oblique nerve-roots at the lower segments of the cord; but the cases of paralysis met with at the Blackwall Tunnel have been very few and trivial compared with those which have been recorded at such works as the St. Louis and the Brooklyn Bridges. By far the most common symptom is pain in the extremities known by the men as "bends.' The pain usually affects the legs, and principally the parts about the knees; the pain may be so severe that it interferes with the use of the limbs to an extent that may simulate paralysis. The theory that the symptoms are due to the giving off of the excess of dissolved gases after removal of the abnormal pressures is the only one considered by the author to be tenable, and this is the foundation of his treatment; for he states that "there is one treatment that should immediately be resorted to —namely, recompression. In order that this may readily be done, without the necessity of conveying the patient down lifts or ladders to the ordinary air locks, a medical air lock should be constructed in a convenient situation. This may be easily done by having an ordinary boiler fixed horizontally [such as that represented on p. 223 of this volume[1]], and a door fitted at one end. If this be divided by a diaphragm provided with a door, the outer chamber serves as a lock whereby the inner can be entered or left without lowering the pressure. When this is provided with bunks, electric light, and the requisite air cocks and connections, we are in possession of a very appropriate medical lock. The patient should be placed in this and the pressure rapidly raised until the pain or other symptom is alleviated. . . . The exit should be effected slowly—in fact,

[1] We are indebted to the kindness of Mr. H. K. Lewis for the loan of the block.

the patient should be allowed to 'leak out' in about forty-five minutes."

Dr. W. Gilman Thompson (*Med. Rec.*, 1894, xlv. 133) quite adopts this same view, that in all instances of caisson disease the main danger lies in the too rapid change of pressure, especially its diminution; and he adds that "one cannot condemn too strongly the practice of allowing the workmen to leave the caisson very rapidly."

If such a chamber as that described should come into daily clinical use for the treatment of emphysema and other conditions where compressed air is found to be useful, then these injunctions to avoid a too rapid change or too long continued a pressure will become increasingly needful.

## Figures and Tables

**Figure f1:**